# Supplementary material for: Ecological and reproductive characteristics of holothuroids Isostichopus badionotus and Isostichopus sp. in Colombia
Source: PLoS One. 2021 Feb 22;16(2):e0247158. doi: 10.1371/journal.pone.0247158 (PMC7899373; doi:10.1371/journal.pone.0247158)
Supplement: S1 Appendix — Including photographs detailing each stage of development. (DOCX) [file pone.0247158.s001.docx]

**Description of the histological characteristics of gonad development**

**Females**

Stage II: growth. In both morphotypes the tubular wall of the gonad is thick and slightly wavy, with an average diameter of 638 + 45 μm (+ SE) in *Isostichopus* sp. and 443 + 127 μm in *I. badionotus*. In the germinal epithelium the growth of abundant basophilic pre-vitellogenetic oocytes can be seen, forming a dark border and with some early vitellogenetic oocytes that begin to migrate towards the lumen (Fig A-1 A, E, I, M).

Stage III: mature. The tubules of the gonad are turgid, their walls have thinned and have very little connective tissue, with an average diameter of 1291 + 88 μm in *Isostichopus* sp. and 1561 + 102 μm in *I. badionotus*. The lumen is filled with densely packed mature oocytes with an average diameter of 98.2 + 1.4 μm (+ SE) for *Isostichopus* sp. and 100.7 + 1.2 μm for *I. badionotus*. In the same way, several follicular cells surrounding the oocyte and the presence of a few pre-vitellogenetic oocytes at the edge of the germinal epithelium are observed (Fig A-1 B, F, J, N).

Stage IV: partly spawned. During spawning not all tubules release their gametes at the same time. In a partial spawning it is observed that the same gonad contains spawned and not spawned tubes. The gonadal tubules lose turgidity, their walls are still thin, and some undulations reappear, the average diameter of the gonad tubes in *Isostichopus* sp. was 943 + 73 μm and in *I. badionotus* 1461 + 88 μm. The presence of abundant mature oocytes and a few in different states of deterioration dispersed in the lumen is observed interspersed with empty areas and some phagocytes, which are larger than the oocytes (Fig A-1 C, G, K, O).

Stage V: spawned. The walls of the tubules increased their thickness and number of undulations, the average diameter of the tubes in *Isostichopus* sp. was 410 + 44 μm and in *I. badionotus* 599 + 80 μm. The lumen was practically empty, except for some remains of mature vitellogenetic oocytes, many of which lost their characteristic shape and the phagocytes were evident (Fig. A-1 D, H, L, P).


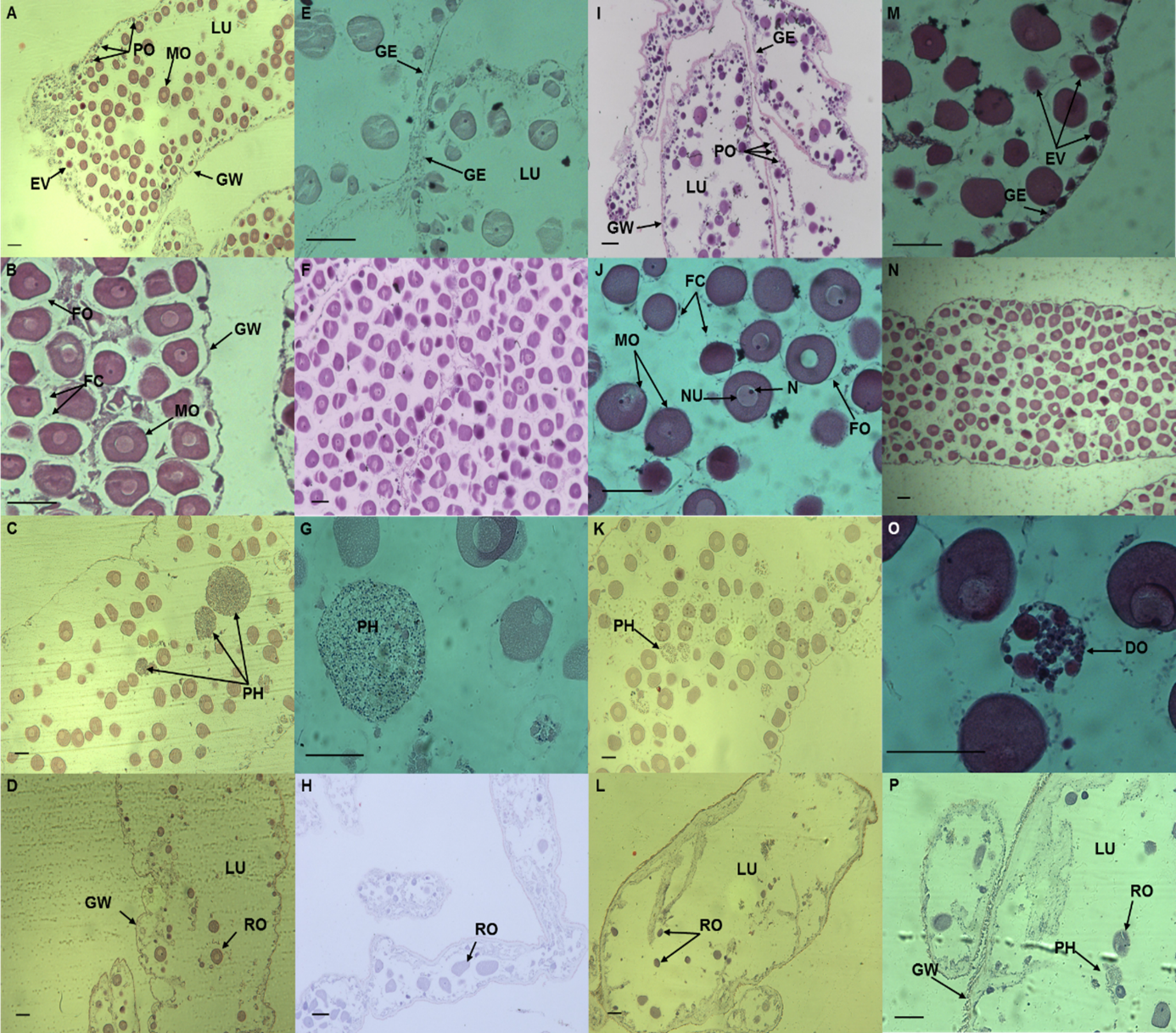


Fig A-1. Microscopic characteristics of different maturity stages in ovary. The figures on the left **(A to H)** refer to *Isostichopus*. sp., figures on the right **(I to P)** refer to *I*. *badionotus*. Stage growing II **(A, E, I, M)**; Stage mature III **(B, F, J, N)**; Stage partly spawned IV **(C,G,K,O)**; Stage spawned V **(D,H,L,P)**; PO: previtellogenetic oocyte, EV: early vitellogenetic oocyte, GW: gonad wall, FO: follicles, FC: follicular cells, NU: nucleus, NC: nucleolus, MO: mature oocyte, PH: phagocytes, RO: remnant oocyte, GE: germinal epithelium, LU: lumen, DO: deteriorated oocyte. The bar indicates 100 µm.

**Males**

Stage II: growth. One of the main characteristics observed in the growth of the testes were the numerous invaginations of the germinal epithelium, which were densely populated by a layer of spermatocytes strongly stained with hematoxylin that formed columns inside the lumen. Likewise, the presence of some mature spermatozoa within the lumen was observed (Fig A-2 A, E, I, M). The average diameter of the tubules of the male gonad in *Isostichopus* sp. was 363 + 28 μm and in *I. badionotus* 634 + 40 μm.

Stage III: mature. The walls of the testicle tubes are thin, and invaginations were reduced or absent, the average diameter of the tubules in *Isostichopus* sp. was 499 + 44 μm and in *I. badionotus* 715 + 21 μm. The interior of the lumen is densely populated with mature spermatozoa that are strongly stained with hematoxylin and several layers of spermatozoa are distinguished with different intensity of staining and in the germinal epithelium it is possible to observe a thin layer of spermatocytes (Fig A-2 B, F, J, N).

Stage IV: partly spawned. The walls of tubules that have experienced spawning appear wrinkled and their walls have begun to thicken. The average diameter of the tubules in this stage for *Isostichopus* sp. was 345 + 23 μm and 502 + 36 μm for *I. badionotus*. In the lumen there are large empty spaces interspersed with small patches of remnant spermatozoa (Fig 4C, G, K, O).

Stage V: spawned. The average diameter of the tubules of *Isostichopus* sp. was 199 + 63 μm and 394 + 34 μm for *I. badionotus*. The walls of the tubules are thick and a little wrinkled, the interior of the lumen is practically empty except for a few remaining sperm (Fig A-2 D, H, L, P).

It should be noted that in many of the partial and totally spawned gonads of both females and males, it was common to see patches composed of dark brown tubules with a pasty texture and easy disintegration to the touch. Histology revealed the presence of abundant connective tissue dispersed inside the lumen, mixed with gametes in an advanced state of deterioration and abundant phagocytes.


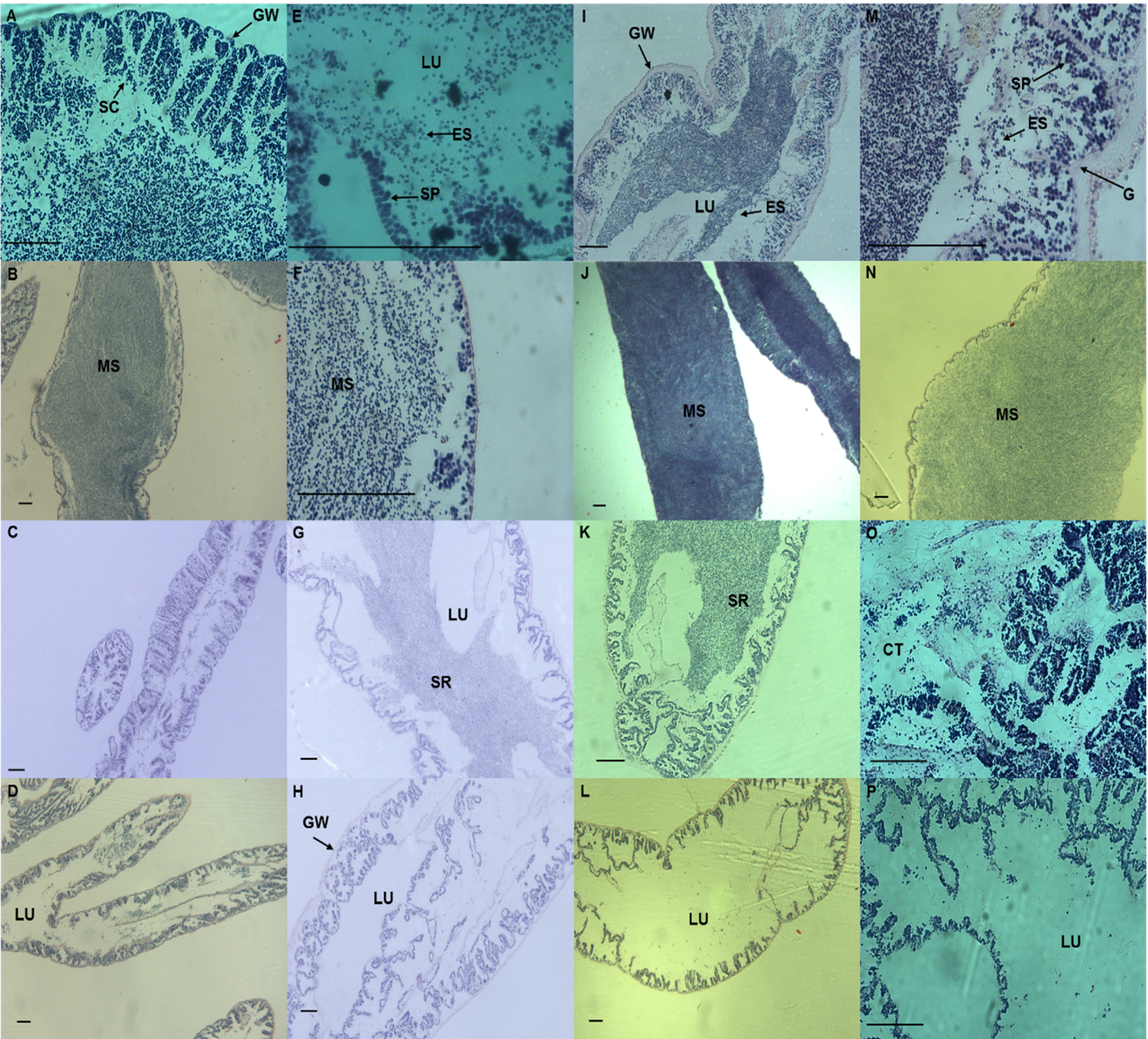


Fig A-2. Microscopic characteristics of different maturity stages in testis. The figures on the left **(A to H)** refer to *Isostichopus*. sp., figures on the right **(I to P)** refer to *I*. *badionotus*. Stage growing II **(A, E, I, M)**; Stage mature III **(B, F, J, N)**; Stage partly spawned IV **(C, G, K, O)**; Stage spawned V **(D, H, L, P)**; GW: gonad wall, GE: germinal epithelium, LU: lumen, SP: spermatocytes, SC: spermatocytes columns, ES: early spermatozoa, MS: mature spermatozoa, SR: spermatozoa remnant, CT: conjunctive tissue. The bar indicates 100 µm.
