# Supplementary material for: Ecological and reproductive characteristics of holothuroids Isostichopus badionotus and Isostichopus sp. in Colombia
Source: PLoS One. 2021 Feb 22;16(2):e0247158. doi: 10.1371/journal.pone.0247158 (PMC7899373; doi:10.1371/journal.pone.0247158)
Supplement: S1 File — (DOCX) [file pone.0247158.s002.docx]

S1 Fig. Relationship between length and fecundity in *Isostichopus badionotus* (*n* = 22)

S2 Fig. Relationship between body wall weight and fecundity in *Isostichopus badionotus* (*n* = 22)

S3 Fig. Relationship between length and fecundity in *Isostichopus* sp. (*n* = 45)

S4 Fig. Relationship between body wall weight and fecundity in *Isostichopus* sp. (*n* = 45)
