# Supplementary material for: Ecological and reproductive characteristics of holothuroids Isostichopus badionotus and Isostichopus sp. in Colombia
Source: PLoS One. 2021 Feb 22;16(2):e0247158. doi: 10.1371/journal.pone.0247158 (PMC7899373; doi:10.1371/journal.pone.0247158)
Supplement: Attachment — Submitted filename: Acosta_etal_PLoSOne_review_Feb2020.pdf [file pone.0247158.s003.pdf]

REVIEW OF PONE-D-20-04776 "Ecological and reproductive characteristics of holothuroids *Isostichopus badionotus* and *Isostichopus sp.* native to the Caribbean coast of Colombia."

REVIEWED BY Jason Hodin

This is a well-carried out study comparing the reproductive biology of two commercially-exploited sea cucumber species (formerly considered a single species) from the Caribbean. Given the massive over-exploitation of many sea cucumber species globally, studies such as this one are important to allow managers to "get ahead of the curve" and institute sound management practices and regulations on the emerging fisheries of currently viable wild populations of sea cucumbers (as with other marine species of commercial interest). The current study is thus welcome in this regard, and is certainly suitable for publication in *PLoS One*.

I do have some significant issues with the data presentation, including the way in which the statistics are presented. I also had some concerns with the literature review in the Discussion, and believe the supplemental information should be integrated with the main text. In addition to these concerns, I have a series of relatively minor comments that I believe the authors should address before re-submitting.

Major comments

1) I believe that the statement on availability of data is insufficient. My understanding is that the authors cannot simply state that the data will be made freely-available, they need to either place those data into a public archive at the the time of publication, or soon thereafter (giving the source in their manuscript for where readers can –or will soon be able to– find the data). The data availability statement should thus be more specific than the generic one given. I ask the editor or editorial staff to please clarify the data availability policy for the authors so that they can rectify this before resubmitting.

2) I have also answered in one of the review questions that "I don't know" if the statistics were properly carried out. That is because I would like to see more details on the statistics presented. Specifically, the authors do not give the *N*'s and degrees of freedom for any of their analyses. Typically, this information is mentioned as subscripts for the *F* (and comparable) statistics.

So (to give a made up example), instead of F=1.005 the authors should write $F_{2,25}$=1.005, which would communicate the degrees of freedom of the analysis (in this case 2) and the within group degrees of freedom (25, which is typically the number of observations within groups).

I would insist that the authors go back through and use this kind of notation or otherwise state the number of observations in each analysis. As is, the statistics are impossible to evaluate. Furthermore, I generally object to results being stated as simply "significant" or "non-significant" without giving further details. Presumably the authors are using P<0.05 as their threshold for "significance" (though this is never stated in the manuscript), but even so, there is potentially a big difference between a P-value of 0.06 and one of 0.5. With little additional effort and space, the authors can sufficiently document the statistics underlying their conclusions throughout.

In the "Minor Comments" section below, I point out some specific instances where I noticed the need for additional statistical documentation. Please note, though, that collectively, what I see as the current insufficient documentation of statistics throughout the manuscript is a Major Comment of mine.

3) Line 150, 165, 176, 185: Confidence intervals around the L50 and W50 values would be helpful for readers and presumably for managers. The spread around the predicted curve is pretty broad. Is there a way to have (for example) 95% c.i. estimates for Figs C, D, G & H? I feel like some kind of statistical test is needed here, since the comparison between the species is stated to be a "clear difference" on Line 313. I don't know how the authors can conclude that there is a "clear difference" without testing for it statistically.

4) Line 205-7. Can the authors do a statistical test for (or otherwise model) the association between gonad index and the environmental parameters? It would be nice to have an objective analysis of what % of the yearly variation in GI could be explained by variations in temp salinity and organic matter. This is especially important since the authors discuss these associations at some length (Lines 329-342) and even mention it again in the short conclusion section (Lines 352-353).

5) Discussion: The authors reference several studies of *I. badionotus* from other locales, including Brasil, Cuba and Panamá. Are the authors certain that these other studies correctly identified the species, given the recent finding that what was formerly considered *I. badionotus* is now considered at least two species (*I. badionotus* and *Isostichopus sp.*)?

6) Supporting Information. I do not see the need to relegate this important information to a supplement. *PLoS One* is an online-only journal and this information on the gonads is important information to both readers interested in the biology of sea cucumbers as well as managers who might attempt to follow the reproductive cycle of these and related cucumbers in order to make management decisions. I would like to see the two Supplemental Figures and the ovary descriptions integrated with the main text.

7) Methodology. There are several places where the Methods were insufficient for me to understand what was done. I have noted several instances in my Minor Comments below, which again, collectively, should be considered a Major Comment of mine.

Minor comments

Line 38 - "unregulated"....but in the next line, it describes environmental enforcement actions. Perhaps the line should be changed to read:

"In Colombia, sea cucumber fishing is carried out illegally on different species, and tends to be mostly unregulated and unquantified [10]. However…"

Line 43 - Is that really paradoxical? Exploitation of local fisherman by the middlemen is probably quite common and the reason is not at all a paradox; the reason is greed and an unregulated marketplace. I suggest, changing "Paradoxically" to something like "Sadly"

Line 50 - change "which they named *Isostichopus sp.*" to "provisionally named *Isostichopus sp.*."

Line 51 - I suggest changing to read:

"Despite fishing pressure on the different morphotypes of *I. badionotus*, as well as on other species of sea cucumbers, research on these exploited species in Colombia has been limited to a few studies on abundance and distribution [2, 16] and reproductive biology [17, 18]."

Line 67: how were animals collected? SCUBA? Also, since you are making calculations about the relative abundance in different habitats (see Lines 119-130), it would be helpful to have a description of the collection effort and search criteria here in the Methods. Finally, how deep did you search? Are you certain there are no more individuals of either species at greater depths than you searched?

Line 71: Please augment the figure legend by describing the different panels. For example, you could say:

"Box in the inset image at lower right is magnified in the inset image at middle right; box in the inset image at middle right is magnified in the main panel."

…or you could label the lower right inset "a" and the middle right inset "b" and then write:

"box in *inset a* is magnified in *inset b*; box in *inset b* is magnified in the *main panel*."

Line 86 & 87: gonad weight and body wall weights are wet weight? Please specify

Also is "body wall weight" the entire cucumber or the entire cucumber minus the gonad or the entire cucumber minus the drained out fluid or…? I am guessing it is the wet weight of the entire cucumber, in which case I would call it "whole body wet weight" rather than "body wall weight", and it could still be abbreviated BW.

I believe that it is the entire body weight, since that would make the gonad index calculation more logical.

The authors refer here to their reference 19, a paper by Chantal Conand in *Bulletin of Marine Science* 1981. I consulted that paper and found that the author did not use the term "body wall weight" — instead she used two weight measurements for the whole animals: what she called "total weight" (TW) and "drained weight" (DW). The former is just what it sounds like- the total wet weight of the intact animal. The drained weight is defined as the total wet weight of the animal "following the opening of the body and the removal of coeliac water."

I suggest more clarity in terminology of measurements, and (more importantly) clear descriptions in the methods of how these measurements were obtained - following the lead of the cited paper by C. Conand.

Line 120, 125: at what depths were the other 2% of *Isostichopus sp*. individuals –and the other 21% of *I. badionotus* individuals– found?

Line 123. Do you have a calculation for what proportion of occupied refuges were occupied by >1 individual?

Line 126. I am not familiar with the meaning of this symbol; I would guess other readers would likewise be unfamiliar.

Lines 128-130. Impressive that the authors had sufficient data to detect a significant difference between 96 and 97% organic matter. Nevertheless, I wonder if that 1% difference

in organic matter is biologically significant in any way?

Line 138. I wonder if "X2" is a universally understood abbreviation? Shouldn't it be a chi symbol and the 2 superscripted (^{2})?

Lines 144-7. The Methods section on Statistics did not mention the Tukey's post-hoc test, which it should. Then you do not need to mention the statistical method here or in Line 171 & line 181 etc.. *F* (and other) statistics throughout the Results should have subscripts listing the degrees of freedom and *N*. Line 145 should list the *P*-value and associated *F* statistics of the "significantly smaller" size of individuals without gonads. I would also appreciate seeing the *P*-values and *F* statistics for the "non-significant" values as well. I think it is worth knowing if the male *versus* female difference had a *P*-value of 0.06 versus (say) 0.5

Line 155. Please add "G and H" before "Length and weight at first maturity for *Isostichopus badionotus*."

Line 181-2. *P*-values and *F* statistics on the weight comparisons please.

Lines 210-212. If the differences for I*. badionotus* are not significant, then how can the authors state that the "the GI of males in both species was consistently lower than that of females"? Also, please give *P*-values and *F* statistics for the "non-significant" values.

Line 220-1 (Fig. 4). I found it very difficult to distinguish the different amounts of grey for the different gonad stages. Please consider redoing this figure with more obvious gradations between the stages, and do this in a manner such that stage 1-6 goes from lightest to darkest shading.

Line 234-235: how was the significance of the increase in organic matter assessed exactly?

Line 253. OK, Now I see how the dry and rainy season is defined. This information needs to go in Line 234 instead. I assume that amounts of organic matter were pooled into these two bins (June-Nov and Dec-May) and then compared?

Lines 264-7. First, why did the authors express their values in the order of magnitude chosen? Wouldn't it make more sense to write:

2.12×10^7

and

1.24×10^8

Furthermore, that is a huge difference in fecundity, that is mostly non-overlapping with respect to the interspecies comparison (the least fecund *I. badionotus* had more oocytes than the average *Isostichopus sp.* This seems worth mentioning and discussing!

Also, I would like to see the gonad weight information reported somewhere. Both species had

comparable gonad indices of about 6-8% at peak maturity. The BW of *I. badionotus* were about 4x higher, but they were about 12x more fecund. Do the authors have an explanation for these values?

I would also like to see fecundity x body size plotted for both species.

Line 270. Please change to read “Average length and weight…” since that is the order those values are reported later in the sentence.

Line 289. “composed mostly of mature adult individuals with low presence of juveniles”

Do the authors attribute this to juveniles existing elsewhere or in more cryptic locations, or that adults are very long lived? Or perhaps another explanation?

Line 325-7. This sentence is confusing, please consider rewriting. The proportion of hermaphroditism in this study was 0.5% in one species and 0% in the other. These are examples of “rare” observations to me. How do these proportions compare to other cucumbers and other aspidochirotids?

Line 328-9 and throughout. Please make sure species names are always italicized.

Line 346-7. It would be nice to have these oocyte sizes (and +/- SD) reported in the Results.

Line 357 - as well as a 120-fold difference in mean fecundity. This should be mentioned and discussed, no?

Supporting Information:

Females section
Check spelling of: “vitellogenic” (it has two “L”s and I believe this is more commonly used that “vitellogenetic”)

Males section
Missing space: “hematoxylin that”

Figures
1) why is this first supplemental figured numbered “S2”?
2) please change “microscopical” to “microscopic” in both S2 & S3 legends
3) please do not capitalize names of structures (e.g., it should be “gonad wall” [lowercase] even if the abbreviation used is GW)
4) all figures helpfully have scale bars but the authors have neglected to tell us how long those scale bars are!!
