# Supplementary material for: Ecological and reproductive characteristics of holothuroids Isostichopus badionotus and Isostichopus sp. in Colombia
Source: PLoS One. 2021 Feb 22;16(2):e0247158. doi: 10.1371/journal.pone.0247158 (PMC7899373; doi:10.1371/journal.pone.0247158)
Supplement: Attachment — Submitted filename: 3rdReview_PONED2004776R3.pdf [file pone.0247158.s007.pdf]

REVIEWER 1 final comments on PONE-D-20-04776R3
Ecological and reproductive characteristics of holothuroids Isostichopus badionotus and Isostichopus sp. in Colombia
====

1. BODY MEASUREMENTS

This was what I wrote in my 2nd review:

> Lines 89-94 should be a single sentence. Therefore, please change Lines 90-92 to read: "...using a flexible 1-m tape measure at the nearest 0.5 cm (this tape contoured the body, so that the total length is the contour length); gonad volume using…"

The authors wrote this in their latest response:

> Answer: Has been changed accordingly. The changes can be seen in the manuscript with corrections in the lines: 108 – 113.

But the authors **did not include the parenthetical I requested** regarding contouring the body with the flexible tape. I will note that Reviewer 2 had a similar comment:

> REV 2: "The process of contouring the measuring tape to the body to measure it's length is not a standard practice for any other animal I can think of. This seems to be a methodological flaw."

To which the authors responded in detail:

> Answer: The measurement was made on the ventral part of the sea cucumber, which is the flattest part of the animal. Measuring from the mouth to the anus. However, due to the ability of these animals to elongate and contract at will, it was decided to standardize the measurements, taking them just after sacrifice by thermal shock in water at 4°C, as this guaranteed that all the animals were in a state of contraction, thus reducing the error. However, in some cases the contracted individuals had a C or S shape for which a flexible metric measuring tape was used to follow the C or S-shape contour of the body. We have deleted this misleading sentence now.

This is not an acceptable solution., As Reviewer 2 notes, the measurement technique employed was unorthodox. Here, the authors have justified that unorthodox technique, but something like the above description (i.e., of the thermal shock treatment preceding measurement, and thus necessitating contour measurements) needs to be included in the Methods. Please add this text before publication.

2. FIGURE EXPONENTS

This was what I wrote in my 2nd review:

> Line 353-356: I still have a problem with the way the exponent figures are listed here. It is very random. Can the authors please make it more easily comparable between species? I suggest rewriting all of these figures as x 10^6

The authors wrote this in their latest response:

> Answer: Has been corrected. The changes can be seen in the manuscript with corrections in the lines: 429 – 432.

Thank you. But the authors appeared to have made an error when changing the figure exponents.

Here is how the prior version read:

> *I. badionotus*, the estimated average fecundity (+ SE) was 74.9 x 10^7 ± 9.5 x 10^7

...and here is how the current version reads (Lines 266-267)

> *I. badionotus*, the estimated average fecundity (+ SE) was 74.9 x 10^6 ± 9.5 x 10^6

The authors seemed to have forgotten to change the values preceding the exponents here as the correct value would appear to be 749 x 10^6 ± 95 x 10^6. Please correct this before publication.

3. LINE 159-160

Please change this sentence to read as follows:

> The sex ratio was not significantly different from 1:1 (*I. badionotus*: n = 100; X2 = 1.4; p > 0.05; *Isostichopus sp.*: n = 158; X2 = 3.3; p > 0.05).

4. LINE 273

Please change this sentence to read as follows:

> ...higher than those reported in neighboring countries \⌋

5. LINE 301 AND FOLLOWING

> This assumption is supported by four facts....

Both I and Rev 2 had problems with this section. I think the problem is that the "four facts" is written as one extremely long sentence.

I would like to see these four facts separated as a numbered list. I.e.,

> This assumption is supported by four facts:
>
> > 1) The total of individuals without gonads possessed all of their internal organs except for the gonad, ruling out the occurrence of auto-evisceration [31];

2) Their average weight and size is greater compared to the weight and size of the smallest individual with gonads...

And so on. If the section is formatted this way (as a separated numbered list) then readers will have a much easier time digesting this information.

6. LINE 384-385

Please change this sentence to read as follows:

> ...especially in species that produce very small oocytes [5, 51], as it is the case with *I. badionotus* (100 µm) and *Isostichopus sp.* (98 µm).

7. SUPPL **S1 Fig (1).docx**

Thank you for including these figures as supplementary material, but when I opened this attachment, I only saw the Figure captions/legend. The four Figures themselves were not present! Please fix this before publication!!
