# Supplementary material for: Ecological and reproductive characteristics of holothuroids Isostichopus badionotus and Isostichopus sp. in Colombia
Source: PLoS One. 2021 Feb 22;16(2):e0247158. doi: 10.1371/journal.pone.0247158 (PMC7899373; doi:10.1371/journal.pone.0247158)
Supplement: Attachment — Submitted filename: Response to Reviewers PLOS ONE.docx [file pone.0247158.s004.docx]

Cordial greetings,

The questions and recommendations related by the reviewers are answered below.

Yours sincerely,

Ernesto Acosta O.

Reviewer #1. Jason Hodin

Major comments

1) I believe that the statement on availability of data is insufficient. My understanding is that the authors cannot simply state that the data will be made freely-available, they need to either place those data into a public archive at the time of publication, or soon thereafter (giving the source in their manuscript for where readers can –or will soon be able to– find the data). The data availability statement should thus be more specific than the generic one given. I ask the editor or editorial staff to please clarify the data availability policy for the authors so that they can rectify this before resubmitting.

*Answer*: Raw data on the reproductive characteristics of the sea cucumbers evaluated in this study can be found in the public repository: https://osf.io/qtac7/

2) I have also answered in one of the review questions that "I don't know" if the statistics were properly carried out. That is because I would like to see more details on the statistics presented. Specifically, the authors do not give the N's and degrees of freedom for any of their analyses. Typically, this information is mentioned as subscripts for the F (and comparable) statistics.

So (to give a made up example), instead of F=1.005 the authors should write F2,25=1.005, which would communicate the degrees of freedom of the analysis (in this case 2) and the within group degrees of freedom (25, which is typically the number of observations within groups).

I would insist that the authors go back through and use this kind of notation or otherwise state the number of observations in each analysis. As is, the statistics are impossible to evaluate. Furthermore, I generally object to results being stated as simply "significant" or "nonsignificant" without giving further details. Presumably the authors are using P<0.05 as their threshold for "significance" (though this is never stated in the manuscript), but even so, there is potentially a big difference between a P-value of 0.06 and one of 0.5. With little additional effort and space, the authors can sufficiently document the statistics underlying their conclusions throughout.

In the "Minor Comments" section below, I point out some specific instances where I noticed the need for additional statistical documentation. Please note, though, that collectively, what I see as the current insufficient documentation of statistics throughout the manuscript is a Major Comment of mine.

*Answer*: Has been changed accordingly to the recommended. All the analyses were reviewed again and some errors were corrected.

3) Line 150, 165, 176, 185: Confidence intervals around the L50 and W50 values would be helpful for readers and presumably for managers. The spread around the predicted curve is pretty broad. Is there a way to have (for example) 95% c.i. estimates for Figs C, D, G & H? I feel like some kind of statistical test is needed here, since the comparison between the species is stated to be a “clear difference” on Line 313. I don't know how the authors can conclude that there is a "clear difference" without testing for it statistically.

*Answer*: In response to the recommendation, L50 and W50 were recalculated for both species using the R statistic, and the 95% confidence intervals were obtained using the BCa (Bias-corrected and accelerated) method. Likewise, on the recommendation of a statistical advisor, the formula for calculating average sexual maturity was adjusted.

The statement that there is a "clear difference" between species in relation to L50 or W50 would be supported by the fact that the confidence intervals constructed in both species do not overlap.

Since P-values or confidence intervals can be used to determine whether there are statistically significant differences, as the results always agree, i.e. if a t-test (which would be the case with the mean difference test) finds a difference between two means, the confidence interval will also show this.

4) Line 205-7. Can the authors do a statistical test for (or otherwise model) the association between gonad index and the environmental parameters? It would be nice to have an objective analysis of what % of the yearly variation in GI could be explained by variations in temp salinity and organic matter. This is especially important since the authors discuss these associations at some length (Lines 329-342) and even mention it again in the short conclusion section (Lines 352-353).

*Answer*: Has been changed accordingly to the recommended

5) Discussion: The authors reference several studies of I. badionotus from other locales, including Brasil, Cuba and Panamá. Are the authors certain that these other studies correctly identified the species, given the recent finding that what was formerly considered I. badionotus is now considered at least two species (I. badionotus and Isostichopus sp.)?

*Answer*: The studies carried out previously in other countries such as Cuba, Panama or Brazil on Isostichopus badionotus carried out a correct identification of the species based on the taxomic characteristics and methods available at that time, however, given that the study concerning the identification and characteristics of this new species Isostichopus sp. was recently published (year 2018) and the area of study in which this research was carried out was limited to a part of the Colombian Caribbean, we do not have sufficient evidence to affirm or deny that the studies carried out previously in other countries would have made a correct identification of the species in the light of the new findings.

6) Supporting Information. I do not see the need to relegate this important information to a supplement. PLoS One is an online-only journal and this information on the gonads is important information to both readers interested in the biology of sea cucumbers as well as managers who might attempt to follow the reproductive cycle of these and related cucumbers in order to make management decisions. I would like to see the two Supplemental Figures and the ovary descriptions integrated with the main text.

*Answer*: Has been changed accordingly to the recommended

7) Methodology. There are several places where the Methods were insufficient for me to understand what was done. I have noted several instances in my Minor Comments below, which again, collectively, should be considered a Major Comment of mine.

*Answer*: Has been changed accordingly to the recommended

Minor comments

Line 38 - “unregulated”....but in the next line, it describes environmental enforcement actions. Perhaps the line should be changed to read:

“In Colombia, sea cucumber fishing is carried out illegally on different species, and tends to be mostly unregulated and unquantified [10]. However…”

*Answer*: Has been changed accordingly to the recommended

Line 43 - Is that really paradoxical? Exploitation of local fisherman by the middlemen is probably quite common and the reason is not at all a paradox; the reason is greed and an unregulated marketplace. I suggest, changing “Paradoxically” to something like “Sadly”

*Answer*: Has been changed accordingly to the recommended

Line 50 - change “which they named Isostichopus sp.” to “provisionally named Isostichopus sp..”

*Answer*: Has been changed accordingly to the recommended

Line 51 - I suggest changing to read:

“Despite fishing pressure on the different morphotypes of I. badionotus, as well as on other species of sea cucumbers, research on these exploited species in Colombia has been limited to a few studies on abundance and distribution [2, 16] and reproductive biology [17, 18].“

*Answer*: Has been changed accordingly to the recommended

Line 67: how were animals collected? SCUBA? Also, since you are making calculations about the relative abundance in different habitats (see Lines 119-130), it would be helpful to have a description of the collection effort and search criteria here in the Methods. Finally, how deep did you search? Are you certain there are no more individuals of either species at greater depths than you searched?

*Answer*: Has been changed accordingly to the recommended

In relation to the question whether it is possible to find individuals of either species at greater depths than those used in this study? the answer is yes. During the doctoral research that generated this manuscript, the issue of density and population status of these sea cucumbers was also addressed. Some dives were made at depths of 25 m, and only specimens of I. badionotus were found, although very rarely. Therefore, we considered that the search depth used in this study (10m) would provide an adequate sample to represent the ecological and reproductive characteristics of both species.

Line 71: Please augment the figure legend by describing the different panels. For example, you could say:

“Box in the inset image at lower right is magnified in the inset image at middle right; box in the inset image at middle right is magnified in the main panel.”

…or you could label the lower right inset “a” and the middle right inset “b” and then write:

“box in inset a is magnified in inset b; box in inset b is magnified in the main panel.”

*Answer*: Has been changed accordingly to the recommended

Line 86 & 87: gonad weight and body wall weights are wet weight? Please specify

Also is “body wall weight” the entire cucumber or the entire cucumber minus the gonad or the entire cucumber minus the drained out fluid or…? I am guessing it is the wet weight of the entire cucumber, in which case I would call it “whole body wet weight” rather than “body wall weight”, and it could still be abbreviated BW.

I believe that it is the entire body weight, since that would make the gonad index calculation more logical.

The authors refer here to their reference 19, a paper by Chantal Conand in Bulletin of Marine Science 1981. I consulted that paper and found that the author did not use the term “body wall weight” — instead she used two weight measurements for the whole animals: what she called “total weight” (TW) and “drained weight” (DW). The former is just what it sounds like- the total wet weight of the intact animal. The drained weight is defined as the total wet weight of the animal “following the opening of the body and the removal of coeliac water.”

I suggest more clarity in terminology of measurements, and (more importantly) clear descriptions in the methods of how these measurements were obtained - following the lead of the cited paper by C. Conand.

*Answer*: The bibliographic reference was modified. The determination of the gonad index was calculated using the body wall weight (BW) defined as weight of the individual without viscera or gonad. This weight (gutted) was chosen, since drained weight may include sediments in the digestive tract and water in the respiratory system, as mentioned by Guzman et al [20].

Likewise, this change in the bibliography generates a modification in the numbering established for the references.

Line 120, 125: at what depths were the other 2% of Isostichopus sp. individuals –and the other 21% of I. badionotus individuals– found?

*Answer*: Has been changed accordingly to the recommended

Line 123. Do you have a calculation for what proportion of occupied refuges were occupied by >1 individual?

*Answer*: Unfortunately, this information was not collected.

Line 126. I am not familiar with the meaning of this symbol; I would guess other readers would likewise be unfamiliar.

*Answer*: Has been changed accordingly to the recommended

Lines 128-130. Impressive that the authors had sufficient data to detect a significant difference between 96 and 97% organic matter. Nevertheless, I wonder if that 1% difference

in organic matter is biologically significant in any way?

*Answer*: taking into account this comment and a similar one suggested by the second evaluator of the manuscript, in reference to the inexactitude of the technique used to measure the percentage of organic matter contained in the sediment and which in turn recommends the elimination of this analysis. Therefore it has been decided to remove the organic matter analysis from the manuscript.

However, as part of the doctoral research of which this manuscript is a part, the analysis of the percentage of organic carbon present in the sediment samples was performed by means of a mass spectrometer and this information has been included in the manuscript.

Line 138. I wonder if “X2” is a universally understood abbreviation? Shouldn’t it be a chi symbol and the 2 superscripted ( 2)?

*Answer*: Has been changed accordingly to the recommended

Lines 144-7. The Methods section on Statistics did not mention the Tukey's post-hoc test, which it should. Then you do not need to mention the statistical method here or in Line 171 & line 181 etc.. F (and other) statistics throughout the Results should have subscripts listing the degrees of freedom and N. Line 145 should list the P-value and associated F statistics of the “significantly smaller“ size of individuals without gonads. I would also appreciate seeing the P-values and F statistics for the “non-significant” values as well. I think it is worth knowing if the male versus female difference had a P-value of 0.06 versus (say) 0.5

*Answer*: Has been changed accordingly to the recommended

Line 155. Please add “G and H” before “Length and weight at first maturity for Isostichopus badionotus.”

*Answer*: Has been changed accordingly to the recommended

Line 181-2. P-values and F statistics on the weight comparisons please.

*Answer*: Has been changed accordingly to the recommended

Lines 210-212. If the differences for I. badionotus are not significant, then how can the authors state that the “the GI of males in both species was consistently lower than that of females“? Also, please give P-values and F statistics for the “non-significant” values.

*Answer*: The data from the IG was reviewed and although differences between females and males only occur in the *Isostichopus* sp.. It was noted that the statistical analysis did not include all the data (only data corresponding to the months of the spawning season were analyzed). Therefore, the statistical analysis was performed again, including the data of all the months evaluated.

Line 220-1 (Fig. 4). I found it very difficult to distinguish the different amounts of grey for the different gonad stages. Please consider redoing this figure with more obvious gradations between the stages, and do this in a manner such that stage 1-6 goes from lightest to darkest shading.

*Answer*: Has been changed accordingly to the recommended

Line 234-235: how was the significance of the increase in organic matter assessed exactly?

Line 253. OK, Now I see how the dry and rainy season is defined. This information needs to go in Line 234 instead. I assume that amounts of organic matter were pooled into these two bins (June-Nov and Dec-May) and then compared?

*Answer*: In effect, the organic matter data were grouped for the periods of dry season (December to May) and rainy season (June to November). However, this analysis was eliminated due to the inexactitude of the technique used to measure organic matter, as already explained in a previous commentary (commentary on Line 128-130).

Lines 264-7. First, why did the authors express their values in the order of magnitude chosen? Wouldn’t it make more sense to write:

2.12 x 107

and

1.24 x 108

Furthermore, that is a huge difference in fecundity, that is mostly non-overlapping with respect to the interspecies comparison (the least fecund I. badionotus had more oocytes than the average Isostichopus sp. This seems worth mentioning and discussing!

*Answer*: Has been changed accordingly to the recommended

Also, I would like to see the gonad weight information reported somewhere.

*Answer*: Has been changed accordingly to the recommended

Both species had comparable gonad indices of about 6-8% at peak maturity. The BW of I. badionotus were about 4x higher, but they were about 12x more fecund. Do the authors have an explanation for these values?

I would also like to see fecundity x body size plotted for both species.

Isostichopus badionotus

Number of eggs

Length (mm)

|  | LENGTH |
| --- | --- |
| 1 | 230 |
| 2 | 240 |
| 3 | 300 |
| 4 | 300 |
| 5 | 310 |
| 6 | 310 |
| 7 | 320 |
| 8 | 320 |
| 9 | 320 |
| 10 | 320 |
| 11 | 320 |
| 12 | 330 |
| 13 | 360 |
| 14 | 360 |
| 15 | 360 |
| 16 | 370 |
| 17 | 370 |
| 18 | 370 |
| 19 | 380 |
| 20 | 450 |
| 21 | 450 |
| 22 | 470 |

| Sea cucumbres | Fecundity |
| --- | --- |
| 1 | 20573380 |
| 2 | 139337893 |
| 3 | 133726971 |
| 4 | 69201370 |
| 5 | 35535838 |
| 6 | 28054609 |
| 7 | 56109219 |
| 8 | 57979526 |
| 9 | 26184302 |
| 10 | 18703073 |
| 11 | 74812291 |
| 12 | 142143354 |
| 13 | 86034135 |
| 14 | 157105812 |
| 15 | 48627989 |
| 16 | 74812291 |
| 17 | 41146760 |
| 18 | 80423213 |
| 19 | 27119456 |
| 20 | 102866901 |
| 21 | 144013661 |
| 22 | 84537889 |

Isostichopus badionotus

Number of eggs

Body wall weight (g)

|  | BODY WALL WEIGHT |
| --- | --- |
| 1 | 450 |
| 2 | 513.6 |
| 3 | 521.6 |
| 4 | 534.3 |
| 5 | 536 |
| 6 | 560.6 |
| 7 | 609.2 |
| 8 | 614 |
| 9 | 614.7 |
| 10 | 631.2 |
| 11 | 637.3 |
| 12 | 730.7 |
| 13 | 748.2 |
| 14 | 756.4 |
| 15 | 774.8 |
| 16 | 799.5 |
| 17 | 802.2 |
| 18 | 825.3 |
| 19 | 864.7 |
| 20 | 873 |
| 21 | 950.1 |
| 22 | 986.3 |

| Sea cucumbers | Fecundity |
| --- | --- |
| 1 | 20573380 |
| 2 | 139337893 |
| 3 | 133726971 |
| 4 | 69201370 |
| 5 | 35535838 |
| 6 | 28054609 |
| 7 | 56109219 |
| 8 | 57979526 |
| 9 | 26184302 |
| 10 | 18703073 |
| 11 | 74812291 |
| 12 | 142143354 |
| 13 | 86034135 |
| 14 | 157105812 |
| 15 | 48627989 |
| 16 | 74812291 |
| 17 | 41146760 |
| 18 | 80423213 |
| 19 | 27119456 |
| 20 | 102866901 |
| 21 | 144013661 |
| 22 | 84537889 |

Isostichopus sp.

Number of eggs

Length (mm)

|  | LENGTH |
| --- | --- |
| 1 | 210 |
| 2 | 280 |
| 3 | 230 |
| 4 | 260 |
| 5 | 190 |
| 6 | 170 |
| 7 | 210 |
| 8 | 170 |
| 9 | 210 |
| 10 | 160 |
| 11 | 170 |
| 12 | 140 |
| 13 | 160 |
| 14 | 200 |
| 15 | 160 |
| 16 | 140 |
| 17 | 210 |
| 18 | 230 |
| 19 | 230 |
| 20 | 150 |
| 21 | 205 |
| 22 | 180 |
| 23 | 190 |
| 24 | 200 |
| 25 | 270 |
| 26 | 180 |
| 27 | 160 |
| 28 | 170 |
| 29 | 190 |
| 30 | 180 |
| 31 | 240 |
| 32 | 230 |
| 33 | 180 |
| 34 | 160 |
| 35 | 160 |
| 36 | 250 |
| 37 | 200 |
| 38 | 160 |
| 39 | 190 |
| 40 | 170 |
| 41 | 180 |
| 42 | 140 |
| 43 | 260 |
| 44 | 190 |
| 45 | 250 |
| 46 | 250 |

| Sea cucumbers | Fecundity |
| --- | --- |
| 1 | 31260694 |
| 2 | 6252139 |
| 3 | 16134552 |
| 4 | 35294332 |
| 5 | 2016819 |
| 6 | 34285922 |
| 7 | 26218646 |
| 8 | 18151371 |
| 9 | 12100914 |
| 10 | 56470931 |
| 11 | 32269103 |
| 12 | 17142961 |
| 13 | 14117733 |
| 14 | 13109323 |
| 15 | 5647093 |
| 16 | 10084095 |
| 17 | 2016819 |
| 18 | 4638684 |
| 19 | 5243729 |
| 20 | 6050457 |
| 21 | 3025228 |
| 22 | 5042047 |
| 23 | 28235465 |
| 24 | 40336379 |
| 25 | 55462521 |
| 26 | 2420183 |
| 27 | 30252284 |
| 28 | 4033638 |
| 29 | 14117733 |
| 30 | 7058866 |
| 31 | 6453821 |
| 32 | 17142961 |
| 33 | 16134552 |
| 34 | 34285922 |
| 35 | 6050457 |
| 36 | 9277367 |
| 37 | 7260548 |
| 38 | 2016819 |
| 39 | 7058866 |
| 40 | 4033638 |
| 41 | 32269103 |
| 42 | 2823547 |
| 43 | 16134552 |
| 44 | 48403655 |
| 45 | 12100914 |
| 46 | 7462230 |

Isostichopus sp.

Number of eggs

Body wall weight (g)

|  | BODY WALL WEIGHT |
| --- | --- |
| 1 | 94.4 |
| 2 | 109 |
| 3 | 109.3 |
| 4 | 115.7 |
| 5 | 119.7 |
| 6 | 119.9 |
| 7 | 131.5 |
| 8 | 144.2 |
| 9 | 152.2 |
| 10 | 154.5 |
| 11 | 161.5 |
| 12 | 164.4 |
| 13 | 167.3 |
| 14 | 168.7 |
| 15 | 172 |
| 16 | 172.4 |
| 17 | 179.9 |
| 18 | 185.8 |
| 19 | 186.5 |
| 20 | 190 |
| 21 | 190.5 |
| 22 | 193.9 |
| 23 | 194.2 |
| 24 | 194.9 |
| 25 | 197.7 |
| 26 | 201.1 |
| 27 | 203.1 |
| 28 | 204.6 |
| 29 | 207.1 |
| 30 | 208.6 |
| 31 | 209.2 |
| 32 | 211.6 |
| 33 | 214.2 |
| 34 | 216.5 |
| 35 | 222.8 |
| 36 | 223.2 |
| 37 | 225.7 |
| 38 | 230 |
| 39 | 238.2 |
| 40 | 246.1 |
| 41 | 252.2 |
| 42 | 257.8 |
| 43 | 265.7 |
| 44 | 267.5 |
| 45 | 287.5 |
| 46 | 328.6 |

| Sea cucumbers | Fecundity |
| --- | --- |
| 1 | 31260694 |
| 2 | 6252139 |
| 3 | 16134552 |
| 4 | 35294332 |
| 5 | 2016819 |
| 6 | 34285922 |
| 7 | 26218646 |
| 8 | 18151371 |
| 9 | 12100914 |
| 10 | 56470931 |
| 11 | 32269103 |
| 12 | 17142961 |
| 13 | 14117733 |
| 14 | 13109323 |
| 15 | 5647093 |
| 16 | 10084095 |
| 17 | 2016819 |
| 18 | 4638684 |
| 19 | 5243729 |
| 20 | 6050457 |
| 21 | 3025228 |
| 22 | 5042047 |
| 23 | 28235465 |
| 24 | 40336379 |
| 25 | 55462521 |
| 26 | 2420183 |
| 27 | 30252284 |
| 28 | 4033638 |
| 29 | 14117733 |
| 30 | 7058866 |
| 31 | 6453821 |
| 32 | 17142961 |
| 33 | 16134552 |
| 34 | 34285922 |
| 35 | 6050457 |
| 36 | 9277367 |
| 37 | 7260548 |
| 38 | 2016819 |
| 39 | 7058866 |
| 40 | 4033638 |
| 41 | 32269103 |
| 42 | 2823547 |
| 43 | 16134552 |
| 44 | 48403655 |
| 45 | 12100914 |
| 46 | 7462230 |

*Answer*: High fecundity is considered as a reproductive strategy, which in the case of sea cucumbers that have limited mobility and whose reproduction is external and density-dependent, would allow them to increase their possibilities of reproduction. Bearing this in mind, one of the possible reasons why I. badionotus is more fecund than Isostichopus sp. is possibly due to the fact that the latter seems to present a more gregarious behavior by sharing shelters and concentrating on more specific habitats (the rocky and shallow bottoms). This would allow it to ensure its reproduction with less effort.

On the other hand, I. badionotus occupies more varied habitats, where individuals are generally more separated from each other, and therefore require a greater effort in gamete production to achieve reproductive success.

Line 270. Please change to read “Average length and weight…” since that is the order those values are reported later in the sentence.

*Answer*: Has been changed accordingly to the recommended

Line 289. “composed mostly of mature adult individuals with low presence of juveniles”

Do the authors attribute this to juveniles existing elsewhere or in more cryptic locations, or that adults are very long lived? Or perhaps another explanation?

*Answer*: We consider that the low presence of juveniles is due to the fact that they have more cryptic habits than the adults and due to their size, they occupy places of more difficult access

Line 325-7. This sentence is confusing, please consider rewriting. The proportion of hermaphroditism in this study was 0.5% in one species and 0% in the other. These are examples of “rare” observations to me. How do these proportions compare to other cucumbers and other aspidochirotids?

*Answer*: Has been changed accordingly to the recommended

Line 328-9 and throughout. Please make sure species names are always italicized.

*Answer*: Has been changed accordingly to the recommended

Line 346-7. It would be nice to have these oocyte sizes (and +/- SD) reported in the Results.

*Answer*: Has been changed accordingly to the recommended

Line 357 - as well as a 120-fold difference in mean fecundity. This should be mentioned and discussed, no?

*Answer*: Has been changed accordingly to the recommended

Supporting Information:

Females section Check spelling of: “vitellogenic” (it has two “L”s and I believe this is more commonly used that “vitellogenetic”)

*Answer*: Has been changed accordingly to the recommended

Males section Missing space: “hematoxylin that”

*Answer*: Has been changed accordingly to the recommended

Figures 1) why is this first supplemental figured numbered “S2”? 2) please change “microscopical” to “microscopic” in both S2 & S3 legends 3) please do not capitalize names of structures (e.g., it should be “gonad wall” [lowercase] even if the abbreviation used is GW) 4) all figures helpfully have scale bars but the authors have neglected to tell us how long those scale bars are!!

*Answer*: Has been changed accordingly to the recommended

Reviewer #2: The manuscript describes results of a study about the reproductive periodicity of two species of sea cucumbers found in Columbian waters. Both species are apparently exploited in illegal fisheries. The study has the potential to support more refined management plans for the two species, such as by informing the planning of seasonal closures and minimum legal size limits.

Generally, the manuscript needed more work on tightening the text and editing out typographic flaws prior to submission. Did all of the co-authors carefully read this manuscript and approve of this version to be submitted? This is mandated by the Vancouver Protocol, and indeed specifically by this journal’s policy. Overall, there are numerous careless errors with the writing (and I don’t just mean English usage). Some paragraphs are comprised of one unwieldy sentence, some careless errors with units and precision, flaws in syntax of citing authors. The job of the scientific reviewers is not to edit the manuscripts in this regard. This should be accomplished by the authorship team.

The Results section is far too wordy, and some of it repeats what has already been stated in the Methods (e.g., ANOVA tests and post-hoc tests). I recommend that the authors trim down the Results section by around 40%.

The authors also used a loss-on-ignition method (line 81) to “determine the content of organic matter” in sediments. This method was borrowed from terrestrial ecologists long ago. Bias of this method for analysing tropical sediments that can contain recent carbonates has been long recognised (Dear 1974, Telek and Marshall 1974, Hirota and Szyper 1975, Morse and Mackenzie 1990, Luczak et al. 1997). The problem is that, some carbon will be lost (as CO2) from the mineral component in addition to the oxidative decomposition of organic matter, leading to an overestimation of organic matter. Although this bias is likely to be fairly consistent among treatment and control samples in the current study, the use of such methods in manuscripts like this can be a detriment to the field of research because other authors will consider publication of the work to be peer-approval of the methods for other studies. I believe that the authors must either remove these results from their manuscript (which would not be a great loss anyway) or re-analyse the sediments in a more correct way that does not lead to overestimation of organic matter.

*Answer:* considering the inexactitude of the technique used to measure the percentage of organic matter contained in the sediment and similarly the comments made by Reviewer #1 regarding the small difference between the percentages of organic matter. It was decided to remove the analysis of organic matter from the manuscript.

However, as mentioned above in Reviewer #1's comments. This manuscript is part of a doctoral research in which the analysis of the percentage of organic carbon present in the sediment samples was also performed. This information was included in the manuscript in order to be able to estimate in some way if there are differences in sediment quality between species or their nutritional value.

Abstract:
Line 13 says holothurians, but elsewhere it says holothuroids. Be consistent and say holothuroid throughout.

*Answer*: Has been changed accordingly to the recommended

According to the authors, Isostichopus sp. is not yet described taxonomically. Is it indeed a separate species or a putative species?

*Answer*: We believe they are two separate species. The breeding tests of the two species do not show a cross between them. And if we are talking about lineages, it would be two lineages by adaptive differences.

Line 15 says “the two bays”. But there are not on two bays in this region.

*Answer*: Has been changed accordingly to the recommended

On line 20 and elsewhere in the manuscript, please replace “presented” with something else such as “had”.

*Answer*: Has been changed accordingly to the recommended

You could say “much lower” on line 21.

*Answer*: Has been changed accordingly to the recommended

On line 25, is it the design or the measures that should not be the same for the two species?

*Answer*: Has been changed accordingly to the recommended

Body Text

Syntax or typo errors on lines 22, 63, 69, 70, 82, 84, 103, 120, 125, 138, 144, 152, 177, 191, 214–216, 221, 240, 254, 261, 285, 296, 299, 323, 328–329, 341.

There are many inconsistencies that need to be fixed. For example, sometimes spaces before and after equal sign but sometimes not.

The first paragraph of the Introduction is one big unwieldy sentence—please revise. Same for first part of second para in Introduction. Other instances in the manuscript should be revised too.

Errors with citation styles: authors should not write “described by [15]”, or “”[18] point out”, or “by [30]”. Name the author(s) in such cases. Fix these throughout the manuscript.

Line 50 says “which they named Isostichopus sp.” but the species has not been named.

Don’t start sentences with numbers. Please rewrite them to start with a word.

*Answer*: Has been changed accordingly to the recommended

On line 85, please describe how the animals were measured? Why was a flexible tape measure used? Was the tape contoured to the body such that the length is the contour length? If so, state this.

*Answer*: Has been changed accordingly to the recommended

Describe the ANOVA analyses better. What factors? Are the factors random or fixed? One-way or two-way ANOVA?

All statistical terms should be italicized, including F, p, K, X2, etc.

*Answer*: Has been changed accordingly to the recommended

The precision used for stating values in the Results section is most often unwarranted, especially when one looks at the error estimate. For body weights and lengths, round to nearest whole numbers.

The methods section already says that ANOVA and post-hoc tests were used. So in the Results, there is no need to say things like “ANOVA results showed…”. Likewise, please don’t have statements such as “The percentage….is shown in Fig 4”.

Lines 205–207 says that the GI values of both species were highest in August, with “This increase coincided with an increase in water temperature, the onset of the rainy season and an increase in organic matter concentrations in the sediment.”. Sorry, but from the Figure, I cannot see a good correlation between organic matter and the GI values. These appear unrelated. Please either analyse this correspondence or reword.

The wording in the Discussion could be tightened.

Don’t use hyphens to indicate a range in data or time periods. Use en dashes in these cases. Also, the en dash replaces the word “to” so it is a grammatical error to write “between XX–XX”.

*Answer*: Has been changed accordingly to the recommended

For the Discussion, the authors have not convincingly proven or shown that temperature, rainfall and runoff are all needed for the animals to spawn during late summer. This would imply that if there was a year without much rain, then the animals would not spawn. Instead, be more circumspect and say warm seawater temperature and/or rainfall.

The graph axes going from December 2015 to January 2017 with only one year of months in between looks very strange. I would suggest to just have the months in the axes, and explain about the pooling of monthly data in the captions.

How does the average fecundity of I. badionotus compare with estimates of fecundity of other species? 124 million oocytes seems like a lot. Please can you compare with values from other studies on other species?

*Answer*: Has been changed accordingly to the recommended
