# Supplementary material for: Ecological and reproductive characteristics of holothuroids Isostichopus badionotus and Isostichopus sp. in Colombia
Source: PLoS One. 2021 Feb 22;16(2):e0247158. doi: 10.1371/journal.pone.0247158 (PMC7899373; doi:10.1371/journal.pone.0247158)
Supplement: Attachment — Submitted filename: Response to the reviewers.docx [file pone.0247158.s005.docx]

Ernesto Acosta O.

Leibniz Centre for Tropical Marine Research (ZMT) GmbH

Fahrenheitstr. 6, 28359 Bremen, Germany

[ernesto77.acosta@gmail.com](mailto:ernesto77.acosta@gmail.com)

Editor-in-Chief

PLOS ONE Journal

August 29, 2020.

Dear Editor and reviewers,

I would like to express my thanks for your time and for the suggested corrections, which have all been addressed and we appreciate that a substantial improvement has been achieved in the manuscript.

Please see the individual responses below and please note that the new line numbers refer to manuscript with Track Changes.

Thank you very much for your kind consideration and would be pleased to provide you with

any additional information,

Kind regards,

Ernesto Acosta.

Reviewer #1

Lines 20-22: please change to read:

“Isostichopus sp. had an average size and weight (193 + 52 mm and 178 + 69 g) and size and weight at first maturity (175 mm and 155 g) that was much lower than I. badionotus (respectively, 324 + 70 mm and 628 + 179 g; 220 mm and 348 g).”

*Answer:* Has been changed accordingly. The changes can be seen in the manuscript with corrections in the lines: 20 – 22.

Line 76: change “divers’ to “snorkelers”

*Answer:* Has been changed accordingly. The changes can be seen in the manuscript with corrections in the line: 84.

Line 78: This would be a good place to mention what the authors mentioned in their response to my original review: that there are likely individuals below 10 meters, but this could not be assessed in the current study.

*Answer:* The information has been included. The changes can be seen in the manuscript with corrections in the lines:87, 88

Lines 89-94 should be a single sentence. Therefore, please change Lines 90-92 to read:

“…using a flexible 1-m tape measure at the nearest 0.5 cm (this tape contoured the body, so that the total length is the contour length); gonad volume using

*Answer:* Has been changed accordingly. The changes can be seen in the manuscript with corrections in the lines: 94 – 106

Line 106: change “states” to “stages”

*Answer:* Has been changed accordingly. The changes can be seen in the manuscript with corrections in the line: 118.


Line 134: please give version of R software used

*Answer:* The information has been included. The changes can be seen in the manuscript with corrections in the line:147


Line 153 and 155: change “had not gonads” to “did not have gonads”

*Answer:* Has been changed accordingly. The changes can be seen in the manuscript with corrections in the lines: 169, 172.

Lines 164, 290, 292, 294, 297, 299, 305: Thank you for providing p-values. But please do not provide that many figures after the decimal place. 3 is sufficient.

So, for example, on line 164 please either write p=0.039 or something like p<0.04.

The K values on lines 164 and 304 are probably also a bit too precise. e.g., on line 164, “6.513” is probably sufficient.

*Answer:* Has been changed accordingly. The changes can be seen in the manuscript with corrections in the lines: 182, 200, 348, 166 and in Table 1.


Line 171: Please change “with” to “that was”

*Answer:* Has been changed accordingly. The changes can be seen in the manuscript with corrections in the line: 190.


Lines 189, 199 and elsewhere if relevant: When listing the F statistics please subscript the degrees of freedom numbers. So, on line 189, the “2,111” should appear as a subscript following “F”

*Answer:* Has been changed accordingly.

Lines 189, 199, 304: p=0.0 is not acceptable. Please write p<0.001

*Answer:* Has been corrected. The changes can be seen in the manuscript with corrections in the lines: 212, 222 and 347.

Lines 228, 258: Change “micras” to “µm”

*Answer:* Has been changed accordingly.

Line 247: change “testicles” to “testes”

*Answer:* Has been changed accordingly.

Line 310: In Table 1, the first row of data is missing cell borders.

*Answer:* Has been corrected.

Line 331: please add to figure legend: “No Stage I individuals were observed in this study (see the text).”

*Answer:* Has been added. The changes can be seen in the manuscript with corrections in the line: 401.


Line 333: change “whose presence” to “such individuals”

*Answer:* This phrase was removed when the size of the results section was reduced, at the request of the second reviewer.

Line 353-356: I still have a problem with the way the exponent figures are listed here. It is very random. Can the authors please make it more easily comparable between species? I suggest rewriting all of these figures as x 10^6

*Answer:* Has been corrected

Line 380: Can the authors please add (perhaps here) a note regarding what they wrote in their response to my original review? Namely, that it’s not likely that the individuals have “low presence” per se, but that due to their size and cryptic habitat, few were observed.

*Answer:* The information has been included. The changes can be seen in the manuscript with corrections in the lines: 470 – 472.

Line 392: please fix this reference where it says “Aspidochrotida [5] and 4”

*Answer:* The number 4 is part of the list of four facts 1), 2), 3), 4), not a reference. The changes can be seen in the manuscript with corrections in the line: 485.

Lines 419-421. interesting. is it possible that hermaphroditism increasing in exploited areas is also possibly due to other co-occurring environmental inputs? In areas with heavy fishing there may also be correlated heavy run-off of land-based pollutants.

*Answer:* We agree that heavy metals can affect the development of gonads and the reproduction of many species. However, during a review of the literature on this topic, no publications were found that related these contaminants to the occurrence of hermaphroditism in sea cucumbers.


Line 472: please correct the word “arts” here

*Answer:* Has been corrected. The changes can be seen in the manuscript with corrections in the line: 570.


Somewhere in discussion: can the authors please somewhere point out what they wrote in their response to my original review, namely:

“we do not have sufficient evidence to affirm or deny that the studies carried out previously in other countries would have made a correct identification of the species in the light of the new findings.”

I believe this is important to mention as it is relevant to their comparisons with prior studies that they made throughout the paper.

*Answer:* Regarding the identification of *Isostichopus* sp. as a new species, the second review suggested that this be reconsidered, as there is still no official scientific recognition of *Isostichopus* sp. as a new species. However, your suggestion was included in the discussion, but the term new species was not used. The changes can be seen in the manuscript with corrections in the lines: 453 – 456.

Figs:

Fig 2 & 5: please make sure that the quality of Figs 2 & 5 are improved when published. The resolution in my version was poor.

*Answer:* Quality has been improved, we did not send the full resolution in the first version, sorry for this. Please note that the PDF version generated during the submission of the manuscript provides very low quality figures, however at the top of each figure you will find a link to access the high quality figure.

Fig 3: panels D,H,L & P still need scale bars

*Answer:* Has been corrected

Fig 6: I’m afraid I still can’t distinguish the grey colors of stages II and III. Can the authors please try one more time to make a visibly different set of grey scale colors for stages II-V in Fig. 6?

*Answer:* we selected new grey scale colors. Please note that the PDF version generated during the submission of the manuscript provides very low quality figures, however at the top of each figure you will find a link to access the high quality figure.

Thank you for including the fecundity x body size plots in you response to my original review. I strongly suggest that the authors include these four plots as supplementary material.

*Answer:* has been included, based on the request of the second reviewer, the histological description of the development of the gonad was moved to the supplementary material section, in order to reduce the size of the results section.

Reviewer #2:

In the title, just write “in Colombia” instead of “native to the Caribbean coast of Colombia”.

*Answer:* The title has been modified according to your suggestion.

Subscript the degrees of freedom after the F when reporting F-values.

*Answer:* The degrees of freedom of each F-value have been written as subscripts. In the manuscript with changes these values can be seen in lines 166, 212, 221, 347.

Okay for the removal of the analysis of organic matter from the manuscript.

Could the authors please double-check all of their references to Figures? For example, line 283 states “an increase in water temperature and the onset of the rainy season ( Fig 5A, B, C)” but that should then be Fig. 5C, D.

*Answer:* All figure references have been checked again and errors found have been corrected (line 316 in the manuscript with changes).

To the first submission, I advised “On line 20 and elsewhere in the manuscript, please replace “presented” with something else such as “had”.” The authors replied “Answer: Has been changed accordingly to the recommended”, yet this remains a grammatical flaw in a couple places in the manuscript.

*Answer:* The word presented has been replaced throughout the document, the changes can be seen in the manuscript with corrections in lines 46, 229, 345 and 548.

You cannot have p = 0.0 in an ecological study such as this. Please fix this (surely the senior authors can help out here).

*Answer:* The p-value has been modified (manuscript with changes line 212, 222 and 347).

Authors were asked in the first review to revise instances in the manuscript where sentences were long and unwieldy. They claim in the response document to have changed this, but there are still clear examples in the manuscript. Just as a glaring example, the second sentence of the Introduction is 68 words long! This is not right for a reviewer to have to make the same recommendation twice.

*Answer:* We are sorry for this, in the revised version many sentences had been shortened already, but obviously not all. This have been revised and corrected again.

Authors were asked in the first review to fix up the precision used for stating values in the Results section. Again, they claim in the response document to have changed this, but there is still unnecessary precision in the manuscript. For example, lines 146, 147, 150, 152-155, 164, 168, 169 and so on…. Again, this is not right for a reviewer to have to make the same recommendation twice.

*Answer:* Have been reviewed and corrected accordingly. In the manuscript with changes these values can be seen in the lines: 154, 155, 159, 162, 163, 166, 168 –.171, 182, 187, 188, 200, 204, 205, 214 – 216, 219, 220, 224, 225.

In the first review, I noted the specific lines with typos or grammatical errors. Many were not fixed up. The authors need to go back over the original submission and fix the errors on those lines.

*Answer:* Have been reviewed and corrected accordingly. In the manuscript with the changes these corrections can be seen in the lines: 68, 75, 97, 99, 151 – 154, 190 206, 217, 218, 227, 233, 314, 315, 318, 347, 350 – 353, 367, 388, 400, 485, 519, 521, 557, 562, 565.

The process of contouring the measuring tape to the body to measure it’s length is not a standard practice for any other animal I can think of. This seems to be a methodological flaw.

*Answer:* The measurement was made on the ventral part of the sea cucumber, which is the flattest part of the animal. Measuring from the mouth to the anus. However, due to the ability of these animals to elongate and contract at will, it was decided to standardize the measurements, taking them just after sacrifice by thermal shock in water at 4°C, as this guaranteed that all the animals were in a state of contraction, thus reducing the error. However, in some cases the contracted individuals had a C or S shape for which a flexible metric measuring tape was used to follow the C or S-shape contour of the body. We have deleted this misleading sentence now.

In the first review, I noted “Don’t use hyphens to indicate a range in data or time periods. Use en dashes in these cases.”. This still appears as an error in some cases.

*Answer:* Has been corrected. The changes can be seen in the manuscript with corrections in the line 519.

Still typos. E.g., line 392 “sea cucumbers of the order Aspidochirotida [5] and 4.”. Line 258 “The bar indicates 100 micras”. Please, all authors, proof-read the version before submission. You are all supposed to approve the submission.

*Answer:* Have been corrected accordingly. The number 4 is part of the list of four facts 1), 2), 3), 4), not a reference. The changes can be seen in the manuscript with corrections in the lines: 485.

The Results section in the original submission was 151 lines. I advised that it was too long and wordy, and recommended to shorten it by around 40%. Instead, the authors have expanded this section and it is now 222 lines. Sorry, this section remains too wordy and I cannot offer my recommendation to have it published like this. So many of the sentences are verbose; there are words that are unnecessary. There are four authors on this manuscript, some of them well published. I am sure some of them could help to revise this section, even if the lead author is a doctoral student. Moreover, it is their duty to do so as authors, according to this journal’s ethics policy, not to mention international codes of ethics including COPE and the Vancouver Protocol.

*Answer:* Compared to the original submission of 151 lines (2106 words), the current results section has now 128 lines (1868 words) of pure text (not counting tables). However, at the request of the first reviewer, new information was included in the results corresponding to the estimation of the variation of the GI as a function of the environmental parameters by means of the Spearman's correlation and the monthly variance of the gonad weight in both species. Part of this new information was introduced by means of two tables.

I like this study. But I am still concerned about the fact that the authors state throughout the manuscript that Isostichopus sp. is a separate species, yet there is no rigorous scientific results to show this. Their response document says only that “We believe they are two separate species.”, but science must be founded on more than beliefs. Without genetic or other morphological evidence to show that they are two distinct species, this should not be reported as such in this manuscript. I am not intending to be difficult here. Rather, this just appears incongruent. Perhaps the authors could reconsider the way in which this putative species is framed in this manuscript?

*Answer:* We welcome your suggestion to change the way *Isostichopus* sp. is addressed in the manuscript and therefore it has been modified, referencing this species as “morphotype *Isostichopus* sp.” (see lines 50 - 54 of the manuscript with track changes).

In the first review, I gave several recommendations and asked for clarification in the Discussion. The author simply replied “Has been changed accordingly to the recommended”, but I cannot see what changes were made and how these points have been addressed. This approach for responding to careful comments by reviewers is grossly insufficient.

*Answer:* We apologize for the manner in which the responses to the first review of this manuscript were presented and hope that the new approach, where each recommendation and question of this new review is addressed, will allow a quick and easy identification of the changes made.
